# A cluster randomised stepped wedge trial to evaluate the effectiveness of a multifaceted information technology-based intervention in reducing high-risk prescribing of non-steroidal anti-inflammatory drugs and antiplatelets in primary medical care: The DQIP study protocol

**DOI:** 10.1186/1748-5908-7-24

**Published:** 2012-03-23

**Authors:** Tobias Dreischulte, Aileen Grant, Peter Donnan, Colin McCowan, Peter Davey, Dennis Petrie, Shaun Treweek, Bruce Guthrie

**Affiliations:** 1 Tayside Medicines Unit, NHS Tayside, Mackenzie Building, Kirsty Semple Way, Dundee DD2 4BF, UK; 2 Population Health Sciences, Quality, Safety and Informatics Research Group, University of Dundee, Mackenzie Building, Kirsty Semple Way, Dundee DD2 4BF, UK; 3 Dundee Epidemiology and Biostatistics Unit (DEBU), University of Dundee, Mackenzie Building, Kirsty Semple Way, Dundee DD2 4BF, UK; 4 University of Dundee, Perth Rd, Dundee DD1 4HN, UK

**Keywords:** Adverse drug event, Non-steroidal anti-inflammatory drug, Antiplatelet, Medication error, Medication review, Decision support systems, Clinical, Stepped wedge, Randomised controlled trial, Primary healthcare

## Abstract

**Background:**

High-risk prescribing of non-steroidal anti-inflammatory drugs (NSAIDs) and antiplatelet agents accounts for a significant proportion of hospital admissions due to preventable adverse drug events. The recently completed PINCER trial has demonstrated that a one-off pharmacist-led information technology (IT)-based intervention can significantly reduce high-risk prescribing in primary care, but there is evidence that effects decrease over time and employing additional pharmacists to facilitate change may not be sustainable.

**Methods/design:**

We will conduct a cluster randomised controlled with a stepped wedge design in 40 volunteer general practices in two Scottish health boards. Eligible practices are those that are using the INPS Vision clinical IT system, and have agreed to have relevant medication-related data to be automatically extracted from their electronic medical records. All practices (clusters) that agree to take part will receive the data-driven quality improvement in primary care (DQIP) intervention, but will be randomised to one of 10 start dates. The DQIP intervention has three components: a web-based informatics tool that provides weekly updated feedback of targeted prescribing at practice level, prompts the review of individual patients affected, and summarises each patient's relevant risk factors and prescribing; an outreach visit providing education on targeted prescribing and training in the use of the informatics tool; and a fixed payment of 350 GBP (560 USD; 403 EUR) up front and a small payment of 15 GBP (24 USD; 17 EUR) for each patient reviewed in the 12 months of the intervention. We hypothesise that the DQIP intervention will reduce a composite of nine previously validated measures of high-risk prescribing. Due to the nature of the intervention, it is not possible to blind practices, the core research team, or the data analyst. However, outcome assessment is entirely objective and automated. There will additionally be a process and economic evaluation alongside the main trial.

**Discussion:**

The DQIP intervention is an example of a potentially sustainable safety improvement intervention that builds on the existing National Health Service IT-infrastructure to facilitate systematic management of high-risk prescribing by existing practice staff. Although the focus in this trial is on Non-steroidal anti-inflammatory drugs and antiplatelets, we anticipate that the tested intervention would be generalisable to other types of prescribing if shown to be effective.

**Trial registration:**

ClinicalTrials.gov, dossier number: NCT01425502

## Background

### Importance of high-risk prescribing of non-steroidal anti-inflammatory drugs and antiplatelets

The quality and safety of prescribing in primary care is an area of increasing concern in the UK and internationally. A number of systematic reviews, including large scale studies conducted in the UK, have demonstrated deficits in the safety and quality of medication use to a degree, which constitutes a public health threat: 3% to 4% of all unplanned hospital admissions are caused by preventable adverse drug events [1,2]. Antiplatelets, non-steroidal anti-inflammatory drugs (NSAIDs), oral anticoagulants, diuretics, and other potentially nephrotoxic agents account for approximately 60% of all preventable admissions.

It is important to distinguish 'high-risk' from 'inappropriate' prescribing, because there will be situations where prescribers and patients are struggling to best manage complex problems for which there is no clearly correct action, and high-risk prescriptions may be justified. However, high-risk prescribing requires systematic management and regular review in order to minimise harm. Effective strategies are therefore needed that enable practitioners to continuously identify, review, and monitor high-risk prescribing [3].

One approach to identifying and reducing high-risk prescribing is the assessment of medication use against explicit measures, thereby highlighting patients who are at risk of adverse outcomes and require a review of benefits and risks. In the UK and internationally, this approach is facilitated by the increasing implementation of electronic patient records in primary care, which enable routine screening for patients at risk of preventable adverse drug events. Such an approach requires, as a minimum, focussing on those quality and safety problems that are strongly linked to patient outcomes based on research evidence [4].

Our previous research has defined and validated a broad set of explicit measures for the assessment of prescribing quality and safety in primary care [5]. In accordance with research evidence, the 'use of antiplatelets and oral NSAIDs in patients at increased risk of gastro-intestinal bleeding' and 'use of oral NSAIDs in patients at increased risk of renal failure' were identified as key priorities for quality improvement in primary care by a Delphi panel of primary care clinicians [5]. We have shown that this prescribing is both common and highly variable between practices, which generally indicates scope for improvement [6].

### Rationale for the Data-driven Quality Improvement in Primary care (DQIP) intervention

There is a large body of research examining changing professional practice to improve the quality of care, much of which has been systematically reviewed [7,8]. A significant proportion of this research relates to improving prescribing [9,10]. Although not all studies show positive effects, there is good evidence for small (~5%) to moderate (11% to 20%) effects of a number of quality improvement strategies. Multi-faceted interventions appear to be more effective than single strategy ones. Strategies shown to be effective include the use of:

1. Feedback of performance data [7], particularly if feedback is prolonged rather than one - off [11].

2. Educational outreach visits, where the evidence is particularly consistent for improving prescribing [8].

3. Interactive educational workshops for continuing professional development [12].

4. Informatics to support clinician decision making and implementation of guidelines [13,14], particularly where it is integrated into existing systems [15].

The broad conclusion of this research is that professional practice can be changed, but that improvements in care are not guaranteed with any strategy and are sometimes short-lived. For example, the PINCER trial recently examined the impact of simple feedback versus pharmacists working in general practices for 12 weeks, delivering education and supporting the review of patients with apparent deficiencies in prescribing or monitoring. Significant reductions in all three primary outcome measures at six months follow-up, but for one (high-risk prescribing of NSAIDs in patients with a history of peptic ulcer), the effect size was smaller and non-significant at 12 months, possibly reflecting that the active intervention was one-off and took place early at the start of the follow-up year [16].

With the spread of electronic medical records and improvements in the information technology (IT) infrastructure, it is increasingly feasible to continuously measure prescribing quality and safety. This allows for timely data feedback and enables practices to identify patients who are currently at risk of preventable drug related harm. On the basis of this information, practices can develop a strategy for systematic patient review and monitor trends of high-risk prescribing at practice level that allows unfavourable trends to be exposed and addressed.

### Trial design

**Figure 1:**
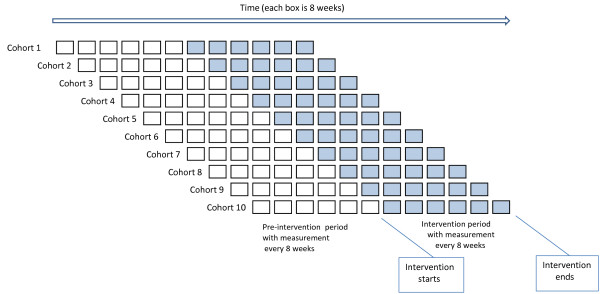
**Illustration of the stepped wedge design**. Each rectangle represents an eight-week time period. Practices are randomised to start the intervention in blocks of four, with measurement of outcome for 48 weeks every eight weeks before the intervention starts and during the 48 weeks of the intervention.

The research team will deliver the DQIP intervention to general practices with the intention of changing professional behaviour, and cluster randomisation at practice level will therefore be used. The trial will use the stepped wedge design [17,18], with all participating practices receiving the DQIP intervention, but randomised to one of ten different start dates and with each practice functioning as their own control in a time series analysis. Practices will only have access to the DQIP tool over the duration of the trial (48 weeks), so that outcome measures during the DQIP intervention will be compared to care prior to it. Practices will be randomised to start the intervention at four weekly intervals with planned gaps to avoid Christmas/New Year and March (the end of the QOF reporting year). The stepped wedge design as it is used in this trial is illustrated in Figure 1.

## Methods

### Participants and settings

The trial will be conducted in volunteer practices in two Scottish health boards, NHS Tayside and NHS Fife. These two Boards provide healthcare for approximately 700,000 patients, with direct responsibility for hospital and community services, and holding the contracts for independent contractors including general practice. Primary medical care is provided by ~130 general practices, and both Boards have a variety of ways in which they seek to influence general practice prescribing, including the use of formularies, guidelines, newsletters, prescribing advice, and the use of primary care pharmacists.

Prescribing advice and primary care pharmacist input varies significantly between the two Boards. In Tayside, there is a greater primary care pharmacy resource in the sense that each practice has a dedicated (usually part-time) primary care pharmacist who works on a mixture of community health partnership and/or Board priorities, and projects agreed with the practice. In Fife, these functions are split between development pharmacists who work at locality level (five localities across three community health partnerships) providing prescribing advice and facilitated discussion of prescribing data, and primary care pharmacists who work in practices, although typically for fewer hours than in Tayside.

In both Boards, general practitioners (GPs) take responsibility for virtually all community prescribing, although nurses, health visitors, and pharmacists are increasingly prescribing some medicines under defined protocols (although this is currently only a small proportion of total prescribing). The intervention therefore targets existing teams of professionals working in general practices in NHS Tayside and NHS Fife.

### Practice inclusion criteria

Practice inclusion criteria include general medical practices in NHS Fife and NHS Tayside using the INPS Vision clinical IT system (n = 78) and agreeing to participate, and practices that agree to have relevant medication related data to be automatically extracted from their electronic clinical information systems

### Practice exclusion criteria

Practices that use GPASS or EMIS clinical IT systems on the date of randomisation will be excluded, because data extraction for the informatics requires the INPS Vision clinical IT system

### Components of the DQIP intervention

**Figure 2:**
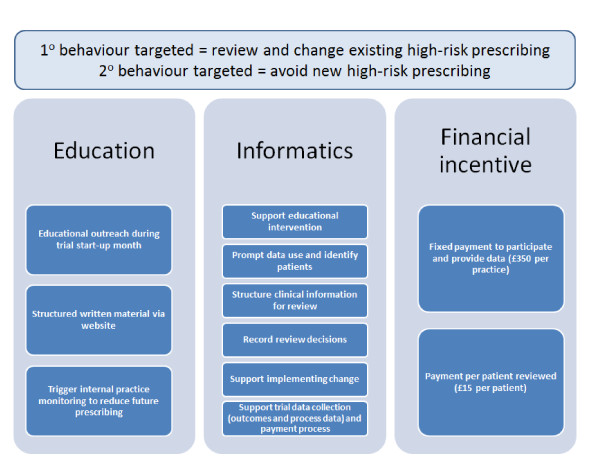
**Illustration of intervention components and desired impact on prescribing behavior**.

The DQIP intervention has three components (Figure 2).

Intervention component one: Informatics tool

The informatics tool is central to the intervention, and will involve the extraction of existing GP clinical data to an NHS Tayside database accessible to the DQIP informatics tool developed by Aridhia (an IT company contracted by NHS Tayside for this purpose). The informatics component will:

1. Summarise numbers of patients affected by high-risk prescribing at practice level based on nine validated measures and one composite measure (see outcome measures). This will allow each practice to monitor trends in high-risk prescribing before and after the start of the DQIP intervention.

2. Identify patients affected by high-risk prescribing for review by the practice, and allow practices to select which patients to target first (e.g., patients triggering particular measures, patients triggering multiple measures).

3. Structure clinical information to facilitate review. All decisions about prescribing and organisation of work around DQIP remain a practice responsibility, including deciding which professional is most suited to do any initial record-based review and any follow-up telephone or face-to-face review.

4. Record review decisions using structured data that supports re-identification of patients if necessary, and to measure trial processes for evaluation.

5. Support reminders to practices to review their data via e-mail reminders.

6. Support the educational intervention (provide general information/summaries of the evidence of risk, links to more detailed reference information, prevalence measurement, and run charts for each practice).

The DQIP informatics tool will additionally collect data for trial process evaluation and record data to trigger practice payment at the end of the trial.

The data held in the NHS Tayside database will be identifiable, and access to it will be controlled in the same way that NHS Tayside controls access to all patient identifiable data (e.g., in Central Vision and other web-based patient information systems), with individual user accounts created at the request of each practice. In NHS Tayside, access will be controlled using the standard lightweight directory access protocol (LDAP) account that primary care clinicians use to access all NHS Tayside clinical databases and tools. In NHS Fife, users will receive a Tayside LDAP guest account with access exclusively granted to the DQIP informatics tool (the exception will be practices in North East Fife which use the Ninewells laboratory and already have Tayside LDAP accounts for Central Vision access). Only practice-based users will have access to identifiable patient information in the informatics tool. For DQIP purposes, a practice-based user is defined as a professional that the practice nominates as an appropriate user, and is expected to include GPs, the practice manager, and (in some cases) primary care pharmacists (who are not practice employees, but often work clinically in practices). With practice permission, the research team will have access to aggregate practice data (prevalence and run charts) during the trial for trial management purposes, and to anonymised patient level data after the trial ends for analysis.

### Intervention component two: Educational outreach

The educational intervention will aim to:

1. Clarify the risk of the targeted prescribing and persuade professionals in participating practices that this prescribing is risky by providing general information with references and links to more detailed information.

2. Persuade professionals that the targeted prescribing is a potential problem in their practice by feeding back prevalence via Tables and run charts.

3. Engage professional/internal motivation to do a 'good job' by persuading professionals that the purpose of the intervention is better patient care, and that this is activity that practices should engage in by appealing to professional values and by creating implicit behavioural norms, including through the use of patient and professional stories.

4. Encourage practices to plan and organise the integration of work associated with reviewing targeted prescribing into practice processes, including the definition of responsibilities of different practice members.

The educational intervention will be delivered via an initial educational outreach visit just before intervention start with an offer of a follow-up visit after approximately three months, and via written material available via the DQIP informatics tool (brief reminders when presenting data, links to more comprehensive documents).

### Intervention component three: Financial incentives to review patients

Historically, trials of this nature have included payment to practices for the time taken to participate in the research, paid via NHS Support Costs. Typically, practices are paid based on an estimate of the time involved. For DQIP, we have chosen to structure the 'NHS Support Costs' as an explicit financial incentive to review patients, with payments modelled on existing general practice 'enhanced service' contracts (specific payments beyond capitation for particular services).

Practices will receive a fixed payment of 350 GBP (560 USD; 403 EUR) for participation and providing data, which is paid before the start of the trial. An important aim of this payment is so that practices agreeing to take part have received a payment while waiting to actually start the intervention; in addition, Practices will receive a payment of 15 GBP (24 USD; 17 EUR) per patient reviewed, where practices earn one payment for each patient reviewed in the year of follow-up rather than payment for every review. This payment will be provided at the end of the trial.

It is critical to note is that there is no financial incentive to change prescribing because this remains completely subject to the clinical judgement of the caring clinician after discussion with the patient. The time line of processes that each cohort of practices will receive as part of the DQIP intervention is shown in Appendix 1.

### Recruitment of general practices

General practices with compatible IT systems in the two health boards will be identified through each board's IT department. All eligible practices (practice manager and all general practitioners in each practice) will receive an email, which informs them of the DQIP trial before formal recruitment starts. This email informs practices of the purpose of the trial and of dates and venues of further information meetings to be held in four different locations across Tayside and Fife. The email will be followed by a formal recruitment letter accompanied by an attractive information sheet that summarises trial processes. The letter will be framed as opt-in to study/opt-out of study and an invitation to request a practice visit by the research team or a phone call to discuss. Practices who do not respond to this letter will receive a phone call. From practices that express an interest, we will obtain informed consent to take part, extract data, and randomised each practice to a start date. Up to three months before the start date in each practice, we will confirm consent to take part and to extract data, as well as obtain a list of professionals who are to have access to the informatics tool.

### Recruitment of patient participants

While the DQIP IT tool (intervention component one) will identify patients who are affected by targeted high-risk prescribing, it will be at each practice's discretion whether and when these patients' prescribing is reviewed and how changes in prescribing (if any) are implemented. This may include writing a letter to the patient, inviting the patient to make an appointment for a phone or face-to-face consultation, or to flag the notes to increase awareness of high-risk prescribing the next time the patient attends. All relevant prescribing decisions and follow-up actions will be recorded in the DQIP IT tool and payment for reviews issued, irrespective of the decisions made (see intervention component three: financial incentives).

### Trial objectives

We hypothesise that the DQIP intervention will reduce rates of high-risk prescribing compared to usual practice. The specific objectives are:

1. To test the effectiveness of the DQIP intervention in reducing the specified primary outcome of a composite measure of high-risk non-steroidal anti-inflammatory drug and antiplatelet prescribing.

2. To test the effectiveness of the DQIP intervention in reducing the specified secondary outcomes of: the nine individual measures constituting the composite; related admissions to hospital; repeat versus new prescribing.

3. To assess the cost-effectiveness of the intervention.

### Sample size

In the pilot practices that targeted high-risk NSAID and antiplatelet prescribing, there was a 40% reduction in this prescribing after one round of feedback and review. This is similar to the short-term reduction in high-risk prescribing achieved in the recently completed PINCER trial [16]. We therefore consider a 20% reduction in high-risk prescribing as measured by our outcome measures plausible (and relatively conservative), and have used this in the power calculation. Power calculations for stepped wedge trials are relatively complex, and three estimates are presented.

### Practice level analysis

The first assumes a practice level analysis, which is the most conservative. Estimated standard deviations are taken from the measured SD in the PINCER trial, and baseline was estimated from a general practice clinical dataset. The number of practices required is estimated to detect a 20% reduction from baseline with power of 80% with a two-sided alpha of 0.05 (Table 1).

**Table 1:** Sample size estimation for practice level analysis

Standard Deviation	Difference 10% to 8%	Effect size	Number of practices
4.0%	2.0%	0.500	34
6.0%	2.0%	0.333	73
7.0%	2.0%	0.286	96

### Patient level analysis

The second estimate assumes that patient level before and after data is available, and 15 practices are estimated to be needed. The third assumes a patient level analysis with time series data available, with three 'before' and three 'after' data points. Power is clearly adequate with approximately 10 practices (Table 2).

**Table 2:** Sample size estimation for patient level analysis with time series data

Number of practices	Effect size	Power
10	0.25	83%
10	0.50	97%
10	0.75	99%
10	> 1.0	> 99%

From this analysis, it is clear that lack of power is not likely to be an issue. Because we know that there are significant differences between the two health boards in the primary care pharmacy resource available to practices, we expect to see differences in the effect size between Boards. Additionally, the literature indicates that interventions of the kind planned often (but not always) have varying effects in larger versus smaller practices. This therefore allows us to plan for well powered within strata analyses, and we plan to stratify randomisation into four strata (the combination of the two binary variables Tayside versus Fife and larger versus smaller) with 10 in each strata.

### Randomisation and allocation concealment

The practice will be the unit of allocation, with practices randomised to an intervention start date using a stepped wedge design. On the assumption that forty practices are recruited, four practices will be allocated to each start date, and randomisation will be stratified in order to ensure balance across time points in terms of:

1. Health board (NHS Tayside and NHS Fife). The rationale is that the underlying organisational context differs, particularly in the way that primary care pharmacy and prescribing advice is organised, and in the focus of prescribing improvement work.

2. List size (larger versus smaller practices). The rationale is that there is evidence that complex interventions may be more difficult to implement in larger practices [19]

3. The stepped wedge randomisation will be computer-generated by the statistician on the project (PD) blinded to practice identity, subsequent to which the core research team (TD, AG, and BG) and participating practices will be informed of their start date.

### Blinding

Due to the nature of the intervention, it is not possible to blind either practices or the core research team (TD, AG, and BG). However, outcome assessment is entirely objective and automated. It is also not possible to blind the analyst because the analysis is a time series in which every practice receives the intervention rather than a comparison between groups. The main analysis will therefore be pre-specified rather than blinded, and any analysis not pre-specified will be clearly identified as such and therefore hypothesis generating/exploratory rather than hypothesis testing/explanatory.

### Outcome measures

The outcome measures used are shown in detail in Tables 3, 4, 5, 6, 7 and the levels of analyses (practice versus patient level) are outlined under 'statistical methods'.

**Table 3:** Primary outcome measure (CPO = Composite prescribing outcome)

Number Risk factor (Denominator)		High-risk prescription definition (Numerator)
CPO	Number of patients with any risk factor listed in PO-measures 1 to 9 (Table 4)	Number of patients with any high-risk prescription listed in PO-measures 1 to 9 (Table 4)

**Table 4:** Secondary outcome measures (PO = Prescribing outcomes)

Number Risk factor (Denominator)		High-risk prescription definition (Numerator)
PO-1	Number of patients with a Read Code for peptic ulceration ever recorded	Number of denominator patients prescribed a traditional oral NSAID^¶ ^or low dose aspirin in the previous eight weeks who have NOT been prescribed a gastro-protective drug in the 12 weeks before, or since the most recent NSAID or aspirin prescription
PO-2	Number of patients aged 75 and over	Number of denominator patients prescribed a traditional oral NSAID^¶ ^in the previous eight weeks who have NOT been prescribed a gastro-protective drug in the 12 weeks before, or since the most recent NSAID prescription
PO-3	Number of patients aged 65 and over prescribed aspirin in the previous 12 weeks	Number of denominator patients prescribed a traditional oral NSAID^¶ ^in the previous eight weeks who have NOT been prescribed a gastro-protective drug in the 12 weeks before, or since the most recent NSAID or aspirin prescription
PO-4	Number of patients aged 65 and over prescribed aspirin in the previous 12 weeks	Number of denominator patients prescribed clopidogrel in the previous eight weeks who have NOT been prescribed a gastro-protective drug in the 12 weeks before, or since the most recent aspirin or clopidgrel prescription
PO-5	Number of patients prescribed warfarin in the previous 12 weeks	Number of denominator patients prescribed a traditional oral NSAID¶ in the previous eight weeks who have NOT been prescribed a gastro-protective drug in the 12 weeks before, or since the NSAID prescription
PO-6	Number of patients prescribed warfarin in the previous 12 weeks	Number of denominator patients prescribed low dose aspirin or clopidogrel in the previous eight weeks who have NOT been prescribed a gastro-protective drug in the 12 weeks before, or since the aspirin or clopidogrel prescription
PO-7	Number of patients with a QOF heart failure code ever recorded	Number of denominator patients prescribed any oral NSAID in the previous eight weeks
PO-8	Number of patients prescribed both a diuretic and an ACE inhibitor/ARB in the previous 12 weeks	Number of denominator patients prescribed any oral NSAID in the previous eight weeks
PO-9	Number of patients with a QOF CKD (stage 3, 4, or 5) code recorded as the most recent CKD code	Number of denominator patients prescribed any oral NSAID in the previous eight weeks
PO-10	**GI risk composite** Number of patients with any risk factors as defined in PO-measures 1 to 6	Number of denominator patients with any high-risk prescription as defined in PO-measures 1 to 6
PO-11	**Renal risk composite** Number of patients with any risk factors in PO-measures 8 to 9	Number of denominator patients with any high-risk prescription as defined in PO-measures 8 to 9

^¶ ^'Traditional' NSAID means all representatives of this class *except *Cox-2 selective agents, such as celecoxib;

**Key: **NSAID = Non-steroidal anti-inflammatory drug; QOF = Quality and outcomes framework; CKD = chronic kidney disease; ACE = angiotensin converting enzyme; ARB = Angiotensin Receptor Blocker

**Table 5:** Secondary outcome measures (Repeated versus new prescribing)

Number	Risk factor (Denominator)	High-risk prescription definition (Numerator)
CPO- Repeated	Number of patients with any risk factor listed in PO-measures 1 to 9 (Table 4)	Number of patients with any high-risk prescription listed in PO- measures 1 to 9 (Table 4) where the patient has received a high-risk prescription in the previous 12 months
CPO-New	Number of patients with any risk factor listed in PO-measures 1 to 9 (Table 4)	Number of patients with any high-risk prescription listed in PO-measures 1 to 9 (Table 4) where the patient has NOT received a high-risk prescription in the previous 12 months

**Table 6:** Secondary outcome measures (AO = Admissions outcomes)

Number	Denominator	Numerator
		Trigger event plus high-risk prescription
**Specific hospital admissions that were preceded by high-risk prescribing**		
AO-1	Patient time with GI risk factors as defined in PO- measures 1 to 6 (Table 4) #	Number of denominator patients admitted with GI bleeding who had any high risk prescription as defined in PO-measures 1 to 6 (Table 4) in the eight weeks before admission
AO-2	Patient time with heart failure as defined in PO-measure 7 (Table 4) #	Number of denominator patients admitted to hospital for HF - exacerbation who had a high-risk prescription as defined in PO-measure 7 (Table 4) in the eight weeks before admission
AO-3	Patient time with renal risk factors as defined in PO-measures 8 to 9 (Table 4)^#^	Number of denominator patients admitted to hospital for acute renal failure or with dehydration or diarrhoea (both defined as potentially inappropriate/ambulatory care sensitive admissions) who had any high-risk prescription as defined in PO-measures 8 to 9 (Table 4) in the eight weeks before admission
**Specific hospital admissions irrespective of high-risk prescribing**		
AO-4	Patient time with GI risk factors as defined in PO-measures 1 to 6 (Table 4)^#^	Number of denominator patients, who are admitted to hospital for GI bleeding
AO-5	Patient time with heart failure as defined in PO-measure 7 (Table 4)^#^	Number of denominator patients, who are admitted to hospital for HF - exacerbation
AO-6	Patient time with renal risk factors as defined in PO-measures 8 to 9 (Table 4)^#^	Number of denominator patients, who are admitted to hospital for acute renal failure or with dehydration or diarrhoea (both defined as potentially inappropriate/ambulatory care sensitive admissions)
**Any cause hospital admissions**		
AO-7	Patient time with GI risk factors as defined in PO-measures 1 to 6 (Table 4)^#^	Number of denominator patients admitted to hospital with any emergency admission
AO-8	Patient time with heart failure as defined in PO-measure 7 (Table 4)^#^	Number of denominator patients admitted to hospital with any emergency admission
AO-9	Patient time with renal risk factors as defined in PO-measures 8 to 9 (Table 4)^#^	Number of denominator patients admitted to hospital with any emergency admission

^# ^The denominator is the time that each patient is defined as having risk factors by virtue of age, disease or co-prescribing in the year before the intervention and the year of follow-up. AO measures 1 to 3 consider specific hospital admissions where patient has had a recent high-risk prescription; AO measures 4 to 6 consider specific hospital admissions irrespective of whether or not the patient has a had a recent high-risk prescription; AO measures 7 to 9 consider all emergency admissions

**Table 7:** Measures of wider impact on NSAID prescribing (VO = Volume outcomes)

VO-1	Total NSAID prescription volume/registered patients in participating compared to non-participating practices
VO-2	Proportion of patients prescribed an NSAID stratified by age in participating compared to non-participating practices

**Key: **NSAID = Non-steroidal anti-inflammatory drug

### Primary outcome measure

Table 3: Primary outcome measure (CPO = Composite of prescribing outcomes)

### Secondary outcome measures

#### Prescribing outcome measures-individual measures

Table 4: Secondary outcome measures (PO = Prescribing outcomes)

##### 'Repeated' versus 'New' prescribing

Table 5: Secondary outcome measures (repeated versus new prescribing)

### Admission outcome measures

Table 6: Secondary outcome measures (AO = admissions outcomes)

### Measures of wider impact on NSAID prescribing

Table 7: Measures of wider impact on NSAID prescribing (VO = volume outcomes)

### Cost-effectiveness

The cost-effectiveness of the intervention will be measured as the cost per reduction in high-risk prescribing and cost per reductions in hospitalisations from an NHS perspective.

### Data collection

#### Prescribing outcomes (PO - 1 to 9)

The primary outcome and prescribing secondary outcomes will be measured in each practice at eight weekly intervals (reflecting that the measure numerators are all for high-risk prescriptions in the last eight weeks). At the time of initial data extraction, eight weekly measures will be constructed for the 48 weeks before the intervention start date.

All outcomes will be measured in the informatics tool using identifiable patient data, but only practices will be able to view identifiable data which will remain in an NHS Tayside controlled database. Access to identifiable data will require explicit practice consent for each individual using the tool, and will be controlled by the NHS Tayside LDAP system used for all clinical systems.

During the study, the core research team will be able to view aggregate practice data (run charts of change over time for each measure) and a summary of practice activity (log-ins, reviews, actions taken). At the end of the trial, fully anonymised data will be extracted from the informatics tool database held by NHS Tayside, and transmitted to the research team using established University of Dundee Health Informatics Centre (HIC) Standard Operating Procedures (SOPs) that require robust anonymisation, transmission using appropriate secure encryption, storage of data on securely encrypted computers, and compliance with Data Protection Act regulations.

### Admission outcomes (AO - 1 to 9)

Scottish Morbidity Recording (SMR01) data on admissions will be linked by HIC to the prescribing data supplied by Aridhia, and provided to the research team under the conditions required by the HIC SOPs described above.

### Statistical methods

The analyses will be implemented under the principle of intention-to-treat and the guiding principles of the ICH E9 for the analysis of randomised controlled trials. All analyses will be carried out under the guidance of the Tayside Clinical Trials Unit, which has UK registration.

The stepped wedge design is essentially a matched design with before and after comparisons for each unit of randomisation. In this case, the unit will be a group of practices where each group is randomised to a particular starting time. In addition to practice level, patient level data will also be collected, and so the analysis is divided into practice level and patient level with the patient level analysis as the primary outcome.

### Practice level analysis

Practices will be characterised by health board, size, age range, proportion of men and women, number of GPs, and training status. The primary outcome of the composite high-risk prescribing measure (Table 3) will be summarised as the mean and standard deviation across practices for before and after the intervention started. Analysis of before compared to after intervention will be carried out using mixed models or multi-level models that account for the correlation of repeated measures (eight-week periods) and stratification by health board in two levels (Tayside and Fife; larger and smaller list sizes). The primary null hypothesis is no difference in the composite before compared to after intervention. A test of the intervention (before versus after) by health board interaction in the model will test whether results differ by health board. Mixed models have the advantage of allowing for missing data while maintaining the principle of intention to treat (ITT). These models can also incorporate adjustment for baseline differences. The practice level analyses will be secondary to the patient level analyses. This will be repeated for all secondary measures (Tables 4 to 7).

### Patient level analysis

The analysis above will be repeated including patient level as well as practice level data. This will be the primary analysis. At the patient level, the primary outcome is binary (yes, no), and so mixed models with binary outcomes will be utilised, i.e., non-linear mixed models. These incorporate the correlation of patients within practices and of repeated measurements over time. At the patient level, descriptive measures will be tabulated such as age, gender, deprivation decile of Scottish Index of Multiple Deprivation (SIMD), number of measures that are relevant to a patient (i.e., the patient meets the denominator definition), as well as the primary and secondary outcomes as percentages.

The mixed model will incorporate fixed terms for intervention (before versus. after), time (eight-week time periods) as well as the correlation of patients within practices and the correlation of repeated measurements over time as random effects. Hence, the analysis will have the element of time series analyses with multiple time points before and after intervention. The primary null hypothesis is no difference in the composite before compared to after intervention as a binary factor. Because patients can be in more than one time period and move in and out of numerators and denominators, the correlation of repeated measures for these individuals will be taken into account in the multilevel modelling. Patients are nested within practices but can be cross-classified with respect to time periods. Initially, this cross classification will be ignored and then the analyses will be repeated with cross-classification terms to assess any differences. These analyses will be repeated for all secondary outcomes.

A formal statistical analysis plan will be documented prior to data lock, and this will form the detailed protocol for analysis. All analyses will be carried out using SAS 9.2 and SPSS v18.

## Discussion

Although IT-based interventions that identify and target patients with high-risk prescribing for review have previously shown to be potentially effective, there is a need for approaches that are more economically sustainable and produce longer-term effects. The DQIP intervention makes use of the existing NHS IT-infrastructure to continuously identify patients with high-risk prescribing and is targeted at existing practice teams rather than relying on additional professional time/resource, which has obvious advantages, especially in the current financial climate.

### Assumptions underlying the design of the DQIP intervention

The design of the DQIP intervention is based on the assumption that in order to be effective in reducing high-risk prescribing of NSAIDs and antiplatelets, then:

The intervention has to be adopted by practices. Adoption is the process by which clinicians and practices are persuaded that the change promoted by the intervention is worth pursuing, that the intervention itself is likely to lead to benefit, and to then ensure that the intervention is implemented. The DQIP intervention aims to support adoption by providing education, an initial fixed payment of 350 GBP (560 USD; 403 EUR), and email reminders to those practices with minimal use of the DQIP tool.

Practices have to reach patients. A necessary condition for the intervention to work is that it is actually delivered to the right patients at the right time. This requires practices to identify suitable patients to deliver the intervention to. If reach is low, then few patients will receive the intervention, and overall effectiveness will also be low irrespective of how 'effective' the intervention is in patients who actually receive it. The DQIP intervention aims to support reach by timely and continuous identification of patients affected by high-risk prescribing, by email -reminders to practices to review their data, and by offering payment of 15 GBP (24 USD; 17 EUR) per patient reviewed.

Practices have to deliver the intervention effectively to patients who are reached. In DQIP, this implies that the way of reviewing and making decisions about high-risk prescribing have to be plausibly effective in reducing the likelihood of adverse drug events from NSAIDs and antiplatelets. The DQIP intervention aims to support effective delivery by structuring clinical information to facilitate review and by alerting practitioners to situations, where patients are re-prescribed high-risk medication against previously documented intentions.

Practices have to maintain these processes for the duration of the trial. A feature of the PINCER study was that 12-month impact of a one-off pharmacy led review was smaller than the six-month effect [20]. In other words, the intervention effect was not fully maintained, either because new patients were prescribed the targeted drugs, or because patients restarted them, or both. Long-term effectiveness requires that the intervention is maintained. The DQIP intervention aims to support maintenance by continuous (rather than one-off) identification of patients affected by high-risk prescribing over the whole duration of the trial, by monitoring whether any improvements in high-risk prescribing are maintained over time (run chart) and alerting practices if this is not the case, and by payment of 15 GBP (24 USD; 17 EUR) per patient reviewed (rather than one-off).

### Design of outcome measures

The DQIP intervention is designed to change prescribing behaviour in primary care and thus, our primary outcome measure and the majority of secondary outcome measures are designed to reflect such behaviour changes.

The core of the intervention is to review existing high-risk prescribing, but we are also hypothesising a reduction in 'new' prescribing, via the triggering of internal practice processes similar to those that appear to have happened with QOF, where stimulating internal monitoring/surveillance have been important mediators of change [21-24]. We do not have any direct control over these internal processes, but will explore their impact by measuring both change in existing prescribing (patients who have received a targeted high-risk prescription in the previous 12 months) and change in 'new' prescribing (patients receiving a targeted high-risk prescription who have not received one in the previous 12 months).

Although not a primary outcome in this trial, the ultimate aim of changing practitioner behaviour is to improve outcomes for patients. However, prescribing is only one among other healthcare process that (in addition to patients' underlying risk factors) may influence outcomes, which implies that interventions focussing on medication use only are unlikely to significantly alter overall mortality or hospital admission rates. Nevertheless, such outcome measures have been used in a number of trials seeking to assess the impact of medication review interventions [25]. We have therefore designed measures that may more specifically capture the potential impact of the DQIP intervention on patient outcomes, i.e., NSAID and antiplatelet specific hospital admissions that are preceded by high-risk prescribing of these agents (AO 1 to 3). Because state-of-the-art assessment of causality and preventability of adverse drug events over the duration of a trial is usually not feasible (clinical judgement by two or more independent experts is usually required), the outcome measures of the type used here may inform the design of similar trials of interventions seeking to improve and measure changes in drug therapy outcomes.

### Rationale for employing the stepped wedge design

The stepped wedge is a relatively novel design for the evaluation of health service interventions. We have chosen this design for two main reasons. First, the staggered entry of practices into the study has logistic advantages in the delivery of the educational outreach visit. If forty practices are recruited, then four practices will start the intervention every four weeks. In contrast, starting all intervention-arm practices at once under a conventional design is not feasible within the resources of our research team. Second, we anticipated a high dropout rate of practices that would have otherwise been randomised to the 'care as usual arm' under a conventional two arm design, because we would not have been able to provide the same financial incentives to these practices, but would have still required these practices' outcome data. We hope that the fact that all participating practices will receive the intervention will facilitate recruitment and reduce drop-out rates [17].

A welcome side effect of the stepped wedge design is that because practices serve as their own controls, the total sample size required for the trial is lower than under a conventional two-arm design (theoretically, a sample size of ten practices would have sufficed for patient level analysis). Recruiting more practices will support the external validity of findings by ensuring that a more representative range of practices participate and to allow examination by strata.

### Anticipated challenges

Despite these advantages, we anticipate a number of challenges. The staggered entry into the trial implies that practices randomised to a later start date will start the intervention up to one year after their initial consent to participate, which implies the risk that initial motivations of practices to take part may no longer be valid at their allocated starting date. We have therefore defined a strategy to maintain the interest of practices while they are waiting to start the DQIP intervention (see Table 8).

**Table 8:** Process for each participating practice

TIME (weeks)	ACTIVITY
While waiting for intervention	Bimonthly newsletter about trial progress; reminder about start date Fixed payment of 350 GBP (560 USD; 403 EUR) (Intervention component 3)
Intervention minus 12	Contact to organise dates for practice training visit; identify who is going to have access to the informatics
Intervention start	Access to informatics turned on (Intervention component 1); Email notification Educational outreach visit (Intervention component 2)^§^ Ask practice to nominate a lead GP as the main practice contact
Intervention plus 4	For practices with minimal use of the tool: Email to lead GP asking for reason and offer further support (IT, clinical, administrative) or practice visits
Intervention plus 8	E-mail newsletter, report on their use of the tool and on changes in their prescribing (positive encouragement irrespective of data, but framed by it). ^§^ For practices with minimal use of the tool: Email to lead GP asking for reason and offer further support (IT, clinical, administrative) or practice visits
Intervention plus 12	Offer second practice visit
Intervention plus 16	E-mail newsletter, report on their use of the tool and on changes in their prescribing (positive encouragement irrespective of data, but framed by it). ^§^ For practices with minimal use of the tool: Email to lead GP asking for reason and offer further support (IT, clinical, administrative) or practice visits
Intervention plus 24	E-mail newsletter, report on their use of the tool and on changes in their prescribing (positive encouragement irrespective of data, but framed by it). ^§^ For practices with minimal use of the tool: Email to lead GP asking for reason and offer further support (IT, clinical, administrative) or practice visits
Intervention plus 32	E-mail newsletter, report on their use of the tool and on changes in their prescribing (positive encouragement irrespective of data, but framed by it). ^§^ For practices with minimal use of the tool: Email to lead GP asking for reason and offer further support (IT, clinical, administrative) or practice visits
Intervention plus 40	E-mail newsletter, report on their use of the tool and on changes in their prescribing (positive encouragement irrespective of data, but framed by it). ^§^ For practices with minimal use of the tool: Email to lead GP asking for reason and offer further support (IT, clinical, administrative) or practice visits
Intervention plus 44	E-mail report, two months to go, encourage final review
Intervention plus 48	E-mail report, one month to go, encourage final review
Intervention plus 52	Thank you, final report on what they did, Payment of 15 GBP (24 USD; 17 EUR) (Intervention component 3)
Intervention plus 4 to 48	Practices are notified of any significant new evidence or guidance relating to targeted high-risk prescribing

^§ ^Encouragement to contact the DQIP team if questions arise or problems occur. The DQIP team will meet any request for further support (IT, clinical, administrative) or practice visits

A further potential problem in stepped wedge designs is contamination between intervention participants and those waiting for the intervention. It is possible that some practices will change their prescribing behaviour as a consequence of being alerted to high-risk NSAIDs and antiplatelet prescribing during recruitment. However, we expect such effects to be mild in comparison to the DQIP intervention because meaningful and sustained reductions in the targeted high-risk prescribing will require systematic and prolonged effort that is unlikely to occur before the DQIP tool is implemented [16].

### Ethical approval

The study has been approved by the Fife and Forth Valley Research Ethics Committee (REC reference 11/AL/0251).

## Abbreviations

NSAID: Non-steroidal anti-inflammatory drug; IT: Information technology; ACE: Angiotensin converting enzyme; QOF: Quality and Outcomes framework; NHS: National Health service; DQIP: Data-driven quality improvement in primary care; ADE: Adverse drug event; GPASS: General Practice Administration System for Scotland; EMIS: Egton Medical Information Systems; LDAP: Lightweight Directory Access Protocol; HIC: Health informatics centre; GP: General practitioner; ITT: Intention to treat.

## Authors' contributions

The DQIP trial is part of a five-year research programme led by BG. The core DQIP research team (TD, AG, and BG) conceived of the research questions and designed the DQIP intervention with contributions by CMcC and PDa. All authors were part of a trial design group led by BG and TD. BG and TD jointly wrote the manuscript, with PD contributing the statistics section. DP has led on the economic analysis. All authors commented on and approved the final version of the manuscript.
